# Prognostic Value of Skeletal Muscle Loss in Unresectable Hepatocellular Carcinoma Treated with TACE-Based Combination Therapy

**DOI:** 10.3390/jcm15135315

**Published:** 2026-07-07

**Authors:** Ningjing Yang, Kaiyu Chen, Chongming Zheng, Puchuang Xu, Junhao Pan, Yuepeng Jin, Gang Chen, Wenhao Hu

**Affiliations:** 1 Department of Radiology, The First Affiliated Hospital of Wenzhou Medical University, Wenzhou 325000, China; yangnj@wmu.edu.cn (N.Y.); pjh1246711344@163.com (J.P.); 2 Department of Hepatobiliary Surgery, The First Affiliated Hospital of Wenzhou Medical University, Wenzhou 325000, China; chen.kaiyu@wmu.edu.cn (K.C.); zcm_1997@163.com (C.Z.); xupuchuangxpc@163.com (P.X.); 3 Zhejiang-Germany Interdisciplinary Joint Laboratory of Hepatobiliary-Pancreatic Tumor and Bioengineering, The First Affiliated Hospital of Wenzhou Medical University, Wenzhou 325035, China; 4 National Health Commission Key Laboratory of Clinical Nutrition and Intervention, The First Affiliated Hospital of Wenzhou Medical University, Wenzhou 325035, China; 5 Department of Interventional Radiology, The First Affiliated Hospital of Wenzhou Medical University, Wenzhou 325000, China

**Keywords:** hepatocellular carcinoma, skeletal muscle loss, sarcopenia, transarterial chemoembolization, combination therapy, prognosis

## Abstract

**Background/Objectives:** Decreases in skeletal muscle mass affect the efficacy of and tumor response to various therapies for hepatocellular carcinoma (HCC). This study aimed to investigate the relationship between changes in skeletal muscle mass during TACE-based combination therapy and tumor response and prognosis. **Methods:** This retrospective study analyzed 306 patients with unresectable HCC, divided into four groups according to the treatment received: TACE alone (n = 133), TACE plus targeted and immunotherapy combination (TACE + T + I, n = 68), TACE plus immunotherapy (TACE + I, n = 52), and TACE plus targeted therapy (TACE + T, n = 53). Skeletal muscle mass was assessed at the third lumbar vertebral level (L3) before treatment and at six months post-treatment using computed tomography (CT) scans. Patients were stratified based on changes in skeletal muscle index (SMI) values. Kaplan–Meier and Cox proportional hazards models were used to compare overall survival (OS) among groups classified by changes in SMI, while tumor response was assessed with univariate and multivariate analyses, using logistic regression analysis. **Result:** A total of 306 patients with unresectable HCC were included in this study. The median OS was 17.0 (0.9–83.0) months in the decreased-SMI group compared with 35.0 (2.0–113.0) months in the non-decreased-SMI group. The overall response rate (CR + PR) was 34.7% in the decreased-SMI group and 53.8% in the non-decreased group. Among different treatment regimens, significant differences in Kaplan–Meier survival curves were observed between the TACE and TACE + T + I groups. In multivariate analysis, decreased SMI remained an independent predictor of poor OS and tumor non-response, whereas a low-SMI pre-treatment was not an independent prognostic factor. Additionally, inadequate nutritional status and compromised liver function were associated with a greater decrease in SMI during comprehensive treatment. **Conclusions:** A decline in skeletal muscle index (SMI) among patients with unresectable hepatocellular carcinoma (HCC) undergoing TACE-based comprehensive treatment is associated with poor prognosis and unfavorable tumor response and is further linked to deteriorating nutritional status and impaired liver function. Monitoring SMI provides an effective approach for assessing overall patient condition and predicting clinical outcomes.

## 1. Introduction

Hepatocellular carcinoma (HCC) is the sixth most prevalent cancer globally and represents the third most common cause of cancer-associated death. It is the major histological subtype of primary liver cancer, comprising approximately 75–85% of cases. Most patients are identified at advanced stages where curative surgical intervention is no longer feasible, and transarterial chemoembolization (TACE) remains the main therapeutic option for patients with unresectable HCC who still have preserved liver function [1]. Numerous studies have demonstrated that, among patients with Barcelona Clinic Liver Cancer (BCLC) stage A or B HCC, TACE achieves objective response rates varying from 36% to 42%, with median overall survival (OS) varying from 19 to 31 months [2]. In advanced HCC, the combination of TACE with immunotherapy and/or targeted therapy has emerged as a predominant local treatment approach [3]. This strategy enhances the tumor immune microenvironment, converting “cold” tumors with poor immune responsiveness into “hot” tumors that elicit stronger antitumor immunity, thereby achieving synergistic therapeutic effects. Randomized controlled trials have shown that combining TACE with targeted and/or immunotherapy in unresectable HCC leads to significant improvements in progression-free survival, overall survival, and quality of life [4,5,6]. 

Moreover, beyond functional reserve and tumor stage, maintaining nutritional balance and physical performance is a critical determinant of long-term outcomes in patients with advanced HCC [7]. Sarcopenia is a clinical syndrome characterized by the progressive loss of skeletal muscle mass and strength, accompanied by reduced physical function [8]. Loss of skeletal muscle mass is a major determinant factor associated with poor survival outcomes and unfavorable tumor response in patients with cancer [7,9,10,11]. In the context of HCC, numerous studies have demonstrated the strong prognostic significance of sarcopenia, showing that it is independently associated with poor OS and progression-free survival (PFS) in HCC patients [12]. This relationship has been consistently observed across different therapeutic approaches, including surgical resection, transarterial chemoembolization (TACE), targeted therapy, and immunotherapy [7,13,14,15].

Muscle loss during treatment may reflect metabolic stress and disease progression. The negative impact of a low SMI on HCC treatment and prognosis may be associated with modulation of the tumor microenvironment, immune function, and cytokine activity [16]. However, existing research has predominantly focused on the association between low baseline muscle mass and overall HCC prognosis, with limited investigation into the prognostic implications of muscle mass decline in patients with unresectable HCC undergoing different TACE-based combination regimens. Furthermore, few studies have systematically evaluated the predictive value of sarcopenia for tumor response and survival outcomes following combination therapy.

This study aimed to assess the association between changes in skeletal muscle mass during TACE-based multimodal treatment regimens and both tumor response and prognosis. In addition, the study aimed to explore the relationships between skeletal muscle loss, nutritional status, and liver function. Our findings highlight a significant correlation between muscle loss during TACE-based combination therapy and both therapeutic efficacy and long-term outcomes, providing important insights for optimizing clinical management in patients with advanced HCC.

## 2. Materials and Methods

### 2.1. Patients

A retrospective cohort study was conducted among patients with hepatocellular carcinoma (HCC) who underwent transarterial chemoembolization (TACE) and systemic therapy at the First Affiliated Hospital of Wenzhou Medical University between January 2017 and December 2024. The inclusion criteria were as follows: (1) patients aged ≥18 years; (2) patients with advanced HCC receiving both TACE and/or systemic therapy within six months; (3) patients with Child–Pugh grade A or B, as well as selected grade C patients enrolled in systemic therapy safety and efficacy trials based on liver function levels; and (4) patients who underwent contrast-enhanced CT or MRI evaluation within one month before and six months after treatment. The exclusion criteria were as follows: (1) absence of abdominal CT scans at baseline and six months post-treatment, and (2) the presence of other malignancies or severe extrahepatic organ diseases. All clinical data were retrieved from electronic medical records. Follow-up for all patients continued until 31 May 2025. This study protocol was conducted in accordance with the ethical principles of the Declaration of Helsinki and was approved by the Institutional Review Board of The First Affiliated Hospital of Wenzhou Medical University [KY2025-RY378]. Informed consent was waived because all data were retrospectively and anonymously analyzed.

### 2.2. Therapeutic Strategies and Response Evaluation

Following a multidisciplinary team (MDT) review, individualized treatment plans were developed for each patient in accordance with clinical practice guidelines, tumor staging, and Child–Pugh classification. Treatment decisions were reached through a shared consensus between healthcare professionals and patients. All TACE procedures were carried out successfully in accordance with the Society of Interventional Radiology (SIR) guidelines [17]. Specifically, the chemotherapeutic agents used during TACE were primarily fluorouracil (400–500 mg/m^2^), epirubicin (40–70 mg/m^2^), and cisplatin (50 mg/m^2^). The embolic agents mainly consisted of Lipiodol (3–5 mL) and gelatin sponge. The dose of chemotherapeutic agents and emulsion of iodized oil were determined through a comprehensive assessment of body surface area, general physical status, and the remnant liver volume. The predominant combined treatment strategies encompassed TACE in conjunction with molecularly targeted drugs or ICIs within a one-month timeframe, along with TACE in combination with molecularly targeted drugs and ICIs. Immune checkpoint inhibitors (ICIs) include inhibitors targeting programmed cell death 1 (PD-1) and programmed cell death ligand 1 (PD-L1) inhibitors. The T group primarily included multi-kinase inhibitors (TKIs) and anti-vascular endothelial growth factor (VEGF) inhibitors.

According to the modified Response Evaluation Criteria in Solid Tumors (mRECIST), treatment response was classified as complete response (CR), partial response (PR), stable disease (SD), or progressive disease (PD) based on findings from contrast-enhanced CT or MRI [18]. Overall survival (OS) was defined as the time interval from the initiation of TACE or systemic therapy to death from any cause or last follow-up.

### 2.3. Imaging Analysis

SliceOmatic software (version 5.0) was used to manually measure fat and muscle areas in the selected tissue regions. The attenuation range for skeletal muscle was defined as −29 to +150 Hounsfield units (HU), whereas intermuscular and subcutaneous fat tissue was delineated using a range of −190 to −30 HU. The skeletal muscle index (SMI) was calculated by dividing the skeletal muscle area at the third lumbar vertebral level (L3, cm^2^) by the square of the patient’s height (m^2^).

### 2.4. Clinical Data

Baseline patient characteristics were recorded, including age, sex, body mass index (BMI), TNM stage, presence of portal vein tumor thrombus (PVTT), and Child–Pugh classification. Laboratory parameters were also included, encompassing coagulation indices, alpha-fetoprotein (AFP) levels, peripheral blood lymphocyte counts, liver function markers (total bilirubin and albumin), HBV-DNA load, and cholesterol levels. Nutritional status was assessed using the Controlling Nutritional Status (CONUT) score, which is calculated based on peripheral blood lymphocytes, albumin, and cholesterol levels [19]. Liver function was evaluated using both the Child–Pugh score and the Albumin–Bilirubin (ALBI) score [20]. BMI was calculated using the standard formula: weight (kg) divided by the square of height (m^2^). The clinical staging of hepatocellular carcinoma (HCC) was determined according to the tumor–node–metastasis (TNM) classification, which incorporates tumor size, number, vascular invasion, lymph node involvement, and distant metastasis. This staging was performed in accordance with the criteria established by the Japanese Liver Cancer Study Group [21]. Based on the guidelines from the Japanese Society for the Study of Hepatology (JSH) for evaluating hepatic sarcopenia, patients were categorized into two groups—Low-SMI and High-SMI—according to their L3-SMI values. The mean cutoff values were 42 cm^2^/m^2^ for males and 38 cm^2^/m^2^ for females [22].

The relative change (%) throughout the treatment process was calculated as follows: ΔSMI = (SMI at 6 months − Baseline SMI)/(Baseline SMI) × 100%. Receiver operating characteristic (ROC) curve analysis identified a cutoff value of −3.34% for ΔSMI [23]. Patients with ΔSMI ≤ −3.34% were assigned to the decreased-SMI group, whereas those with higher values were categorized into the non-decreased-SMI group.

### 2.5. Statistical Analysis

Demographic and disease characteristics were compared between the decreased-SMI group and the non-decreased-SMI group. One-way analysis of variance (ANOVA) was employed to compare continuous variables with a normal distribution among multiple groups. Continuous variables are expressed as mean ± standard deviation (SD), while categorical variables are presented as counts and percentages (%). Parametric data were compared between groups using *t*-tests, while nonparametric data were analyzed with the Mann–Whitney U test. Comparisons of categorical variables were conducted using the Chi-square (χ^2^) test or the Kruskal–Wallis H test. Logistic regression was conducted to determine factors associated with tumor response. Overall survival (OS) was estimated using the Kaplan–Meier method, and corresponding survival curves were plotted. Subsequently, Cox proportional hazards models were constructed to assess statistically significant differences in survival between groups. Statistical analyses were conducted using SPSS version 27.0 (IBM). All statistical tests were two-sided, and *p* values < 0.05 were regarded as statistically significant.

## 3. Results

### 3.1. Patient Characteristics

A total of 678 patients were initially screened, of whom 372 were excluded for various reasons. Ultimately, a total of 306 patients were enrolled and categorized based on a ≥3.34% decrease in SMI at six months post-treatment. Table 1 summarizes the baseline characteristics of 150 patients in the decreased-SMI group and 156 patients in the non-decreased-SMI group. Overall, most clinical parameters were comparable in both groups. Patients in the decreased-SMI group had higher baseline body weight [median 63.75 (57.00–72.00) vs. 60.00 (55.00–67.62) kg, *p* = 0.015] and BMI [23.44 (20.96–25.31) vs. 22.25 (20.13–24.33), *p* = 0.011]. Baseline SMI was also significantly higher in the decreased-SMI group (male: 48.40 ± 8.09 vs. 44.76 ± 7.09 kg/m^2^; female: 39.82 vs. 35.52 kg/m^2^, *p* < 0.001). Furthermore, the percentage of patients presenting with multiple tumors (>3 lesions) was higher in the decreased-SMI group (44.67% vs. 26.28%, *p* < 0.001), as was the incidence of portal vein tumor thrombus (PVTT) (32.00% vs. 16.67%, *p* = 0.002), indicating a greater tumor burden at baseline. There were no significant differences in age, gender, nutritional status, liver function, or clinical stage between the two groups. These baseline imbalances were accounted for in subsequent multivariate analyses to minimize potential confounding effects.

### 3.2. Baseline and Post-Treatment Changes in SMI and Related Factors

**Figure 1:**
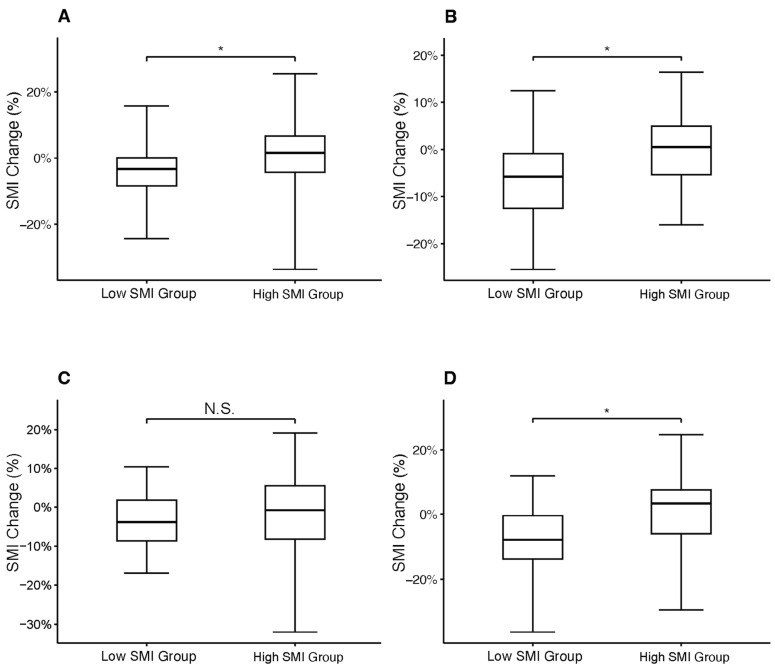
Changes in ∆SMI after 6 months of treatment in patients with low SMI and high SMI. TACE group (**A**), TACE + T group (**B**), TACE + I group (**C**), TACE + I + T group (**D**). ∆SMI, Skeletal Muscle Index; TACE, Transarterial Chemoembolization; I, Immunotherapy; I + T, Targeted Therapy. * *p* < 0.05, N.S., not significant.

We analyzed the relationships between baseline SMI, SMI changes, and treatment regimens. As shown in Table 2, there were no significant differences in baseline SMI or ΔSMI among the four treatment groups. As depicted in Figure 1, patients with low baseline SMI experienced greater muscle loss across all treatment regimens. Specifically, Low-SMI patients demonstrated significantly increased muscle loss (*p* < 0.05) when receiving the TACE (Figure 1A), TACE + I (Figure 1C), and TACE + I + T (Figure 1D) regimens.

**Figure 2:**
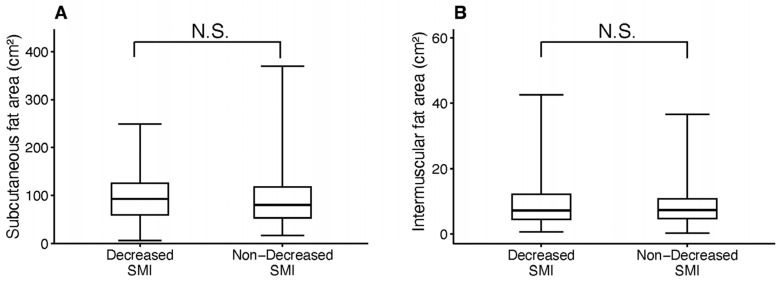
Comparison of fat area and muscle area in CT findings before treatment initiation. (**A**) The median subcutaneous fat area at the umbilical level was 92.96 (6.53–249.1) cm^2^ in the decreased skeletal muscle index (SMI) group and 80.51 (16.5–368.8) cm^2^ in the non-decreased-SMI group. (**B**) The median Intermuscular fat area at the umbilical level was 7.17 (0.65–42.61) cm^2^ in the decreased-SMI group and 7.29 (0.23–36.54) cm^2^ in the non-decreased-SMI group. N.S.: not significant.

Figure 2 illustrates the comparative assessment of baseline intermuscular and subcutaneous fat areas between the decreased-SMI and non-decreased-SMI groups. There were no significant differences in either intermuscular or subcutaneous fat areas between the two groups before the initiation of treatment (Figure 2).

**Figure 3:**
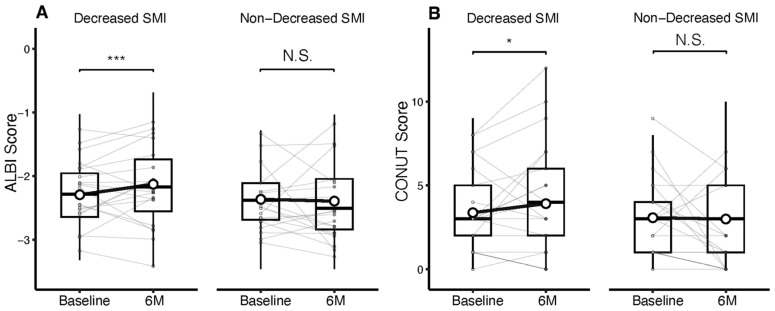
The changes in skeletal muscle mass and related factors in the members of the decreased-SMI (n = 150) and non-decreased-SMI groups (n = 156). (**A**) Controlling Nutritional Status (CONUT) scores increased significantly at 6 months after treatment in the decreased skeletal muscle index (SMI) group. No significant changes were observed in the non-decreased-SMI group. (**B**) The Albumin–Bilirubin (ALBI) scores were significantly decreased at 6 months after treatment in the decreased-SMI group compared to the baseline. No significant changes were observed in the non-decreased-SMI group. * *p* < 0.05; *** *p* < 0.001; N.S.: not significant; 6M: 6 months.

Figure 3 shows the relationship between nutritional status, liver function, and SMI changes in 150 patients with decreased SMI and 156 patients with non-decreased SMI six months after comprehensive treatment. Compared with baseline, both CONUT and ALBI scores significantly increased in the decreased-SMI group at six months, indicating a potential deterioration in nutritional status and liver function during treatment. In contrast, there were no significant changes in the CONUT and ALBI scores in the non-decreased-SMI group at 6 months after treatment. Additionally, we analyzed the AEs during treatment (Table S1). AEs of any grade occurred at a rate of 42.0% in the decreased-SMI group and at a rate of 31.4% in the non-decreased-SMI group. There were no significant differences in AEs depending on whether the SMI decreased during therapy. The major AEs recorded were increased transaminase level, anorexia, vomiting, decreased white blood cell count, anemia, and upper gastrointestinal hemorrhage.

### 3.3. Treatment Response

Table 3 shows the therapeutic effect according to mRECIST criteria based on post-treatment contrast-enhanced CT or MRI after six months of treatment. The overall response rate (CR + PR) was 34.7% in the decreased-SMI group and 53.8% in the non-decreased-SMI group. The disease control rate (CR + PR + SD) was 74.7% in the decreased-SMI group and 85.3% in the non-decreased-SMI group. Overall, the non-decreased-SMI group exhibited significantly better treatment efficacy compared with the decreased-SMI group.

### 3.4. Survival Outcomes and Prognosis Analysis

**Figure 4:**
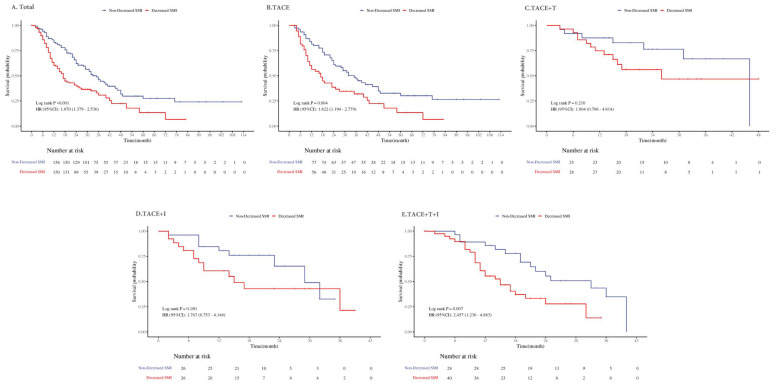
Kaplan–Meier curves for OS stratified by treatment group and SMI change. Decreased-SMI group or non-decreased-SMI group in all patients (**A**) (*p* < 0.01), the TACE group (**B**) (*p* < 0.01), the TACE + T group (**C**) (*p* = 0.21), the TACE + I group (**D**) (*p* = 0.18), and the TACE + T + I group (**E**) (*p* < 0.01). OS, Overall Survival; SMI, Skeletal Muscle Index; ∆SMI, Rate of Change in Skeletal Muscle Mass Index; TACE, Transarterial Chemoembolization; I, Immunotherapy; I + T, Targeted Therapy Combined with Immunotherapy; T, Targeted Therapy.

Clinical outcomes are presented in Figure 4. The median follow-up duration was 32 months (range, 0.9–113.0 months). The median overall survival (OS) was 17.0 months (0.9–83.0) in the decreased-SMI group and 35.0 months (2.0–113.0) in the non-decreased-SMI group. Among the different treatment regimens, significant differences in Kaplan–Meier survival curves between the decreased-SMI and non-decreased-SMI groups were observed in the TACE (Figure 4B; hazard ratio [HR], 1.82; 95% CI, 1.20–2.78) and TACE + T + I groups (Figure 4E; HR, 2.46; 95% CI, 1.23–4.88) (*p* < 0.01).

We analyzed tumor response and OS in patients with unresectable HCC following combination therapy. In multivariate analysis, independent factors significantly associated with OS included a reduction in SMI at six months post-treatment (presence vs. absence: HR 1.60; 95% CI, 1.17–2.19; *p* = 0.03), maximum tumor diameter >5 cm (>5 cm vs. ≤5 cm: HR 1.66; 95% CI, 1.15–2.40; *p* < 0.01), portal vein tumor thrombus (PVTT; yes vs. no: HR 1.45; 95% CI, 1.01–2.07; *p* = 0.04), and tumor number >3 (>3 vs. ≤3: HR 1.88; 95% CI, 1.35–2.62; *p* < 0.01) (Table 4). Additionally, multivariate analysis identified independent factors significantly associated with tumor response: decreased SMI at six months post-treatment (presence vs. absence: OR 0.43; 95% CI, 0.26–0.70; *p* < 0.01), Child–Pugh score ≥7 (≥7 vs. <7: OR 0.53; 95% CI, 0.29–0.95; *p* = 0.03), and tumor number >3 (>3 vs. ≤3: OR 0.35; 95% CI, 0.20–0.60; *p* < 0.01) (Table 5).

## 4. Discussion

This retrospective study explored the association between ΔSMI and both tumor response and OS in a cohort of patients with unresectable HCC receiving TACE-based combination treatments. Our results demonstrated that ΔSMI was independently associated with an inferior therapeutic effect and shorter OS. These results indicate that muscle loss during treatment may reflect metabolic stress and disease progression. Most available studies have focused on single therapeutic modalities, leaving the prognostic value of muscle mass loss during TACE-based multimodal systemic therapy largely unexplored. Our study systematically evaluates dynamic skeletal muscle changes and their association with tumor response and long-term survival in patients with unresectable HCC. These findings highlight the clinical importance of dynamic monitoring and intervention of sarcopenia to optimize outcomes in advanced HCC.

At six months post-treatment, 150 patients exhibited decreased SMI. Notably, there were no significant differences between the decreased and non-decreased groups in baseline nutritional status, liver function, tumor progression, or subcutaneous and intermuscular fat as measured by CT imaging. However, baseline SMI was higher in the decreased-SMI group, suggesting that patients with greater initial muscle reserves experienced more pronounced muscle loss during treatment. There was no significant difference in baseline SMI or ΔSMI among the four treatment regimens. The etiology of skeletal muscle loss in cancer is multifactorial, including reduced physical activity, malnutrition from disease progression and treatment side effects, and increased inflammatory cytokine expression [23,24]. TACE-induced upregulation of PD-1 and PD-L1 expression, which promotes tumor antigen release and pro-inflammatory cytokine secretion, potentially exacerbating muscle loss in Low-SMI patients [25]. Comparative analyses revealed that Low-SMI patients experienced greater muscle loss during TACE, TACE + I, and TACE + I+T therapies. These differences may be explained by the distinct therapeutic mechanisms involved. However, in response to TACE + T treatment, the Low-SMI and High-SMI groups experienced similar levels of muscle loss. This may be related to the inhibitory effects of targeted therapy on the PI3K/AKT/mTOR pathway [26]. Activation of this pathway promotes muscle growth. mTOR, a key regulator of muscle protein synthesis, exerts its effects primarily through the mTORC1 complex, which controls protein synthesis, cell growth, metabolism, and autophagy by phosphorylating downstream targets [27]. Inhibition of this pathway can impair muscle protein synthesis and so could have contributed to the more pronounced muscle loss observed in the High-SMI group.

Previous studies have consistently demonstrated the prognostic significance of skeletal muscle loss across various treatment modalities for HCC, curative hepatectomy combined with adjuvant TACE [28], ICIs [29,30], and TKIs [31]. Uojima et al. identified low skeletal muscle mass as a predictor of reduced OS in patients receiving lenvatinib [32,33], while Kobayashi, Ozbay, and Fujita et al. reported that rapid muscle depletion independently correlated with poor prognosis following transarterial therapy [34,35,36,37]. Similarly, Jin et al. demonstrated that longitudinal declines in skeletal muscle predicted inferior outcomes in unresectable HCC treated with PD-L1 inhibitors plus anti-VEGF antibodies or TKIs [38]. These findings are consistent with our results.

In our cohort, low baseline SMI was not significantly associated with treatment efficacy. However, achieving ORR was significantly correlated with reduced SMI at six months post-treatment. Patients experiencing significant SMI decline following TACE or TACE + I had poorer treatment outcomes, particularly in the TACE monotherapy group. No significant differences were observed in the TACE + T or TACE + T+I groups, likely due to smaller sample sizes, treatment selection bias, and variations in drug combinations. While a low-SMI pre-treatment has been identified as an independent risk factor for recurrence and mortality in HCC [7,39], its prognostic relevance may diminish in unresectable or metastatic disease, where progressive muscle loss is a stronger predictor of survival. Our multivariate analysis confirmed that six-month ΔSMI is a robust independent predictor of OS, whereas baseline SMI does not independently predict outcomes in unresectable HCC, consistent with previous reports [40].

Dynamic monitoring of muscle mass is therefore clinically important for identifying higher-risk individuals and optimizing nutritional and therapeutic interventions. Skeletal muscle cells express major histocompatibility complex molecules and present antigens to T cells; insufficient muscle mass may impair T cell-mediated tumor immunity and limit the response to ICIs [16]. Skeletal muscle also secretes multiple bioactive factors that regulate the tumor microenvironment [41]. IL-15, for instance, promotes tumor immunity via CD8+ T cells and NK cells, key targets of PD-1 or PD-L1 therapy [42]. Reduced muscle mass decreases IL-15 production, impairing NK cell development and survival, potentially facilitating immune escape [43]. The underlying mechanisms linking muscle loss to impaired immune regulation and unfavorable outcomes in HCC require further investigation.

Furthermore, multivariate analysis identified tumor number (>3), maximum tumor diameter (>5 cm), and PVTT as independent prognostic factors for overall survival in patients receiving combination therapy, corroborating previous findings [35]. Patients with decreased SMI also showed significant increases in ALBI and CONUT scores at six months, indicating that liver dysfunction and malnutrition may contribute to treatment-related muscle loss. Branched-chain amino acid (BCAA) supplementation can restore albumin levels, decrease intramuscular fat deposition, preserve skeletal muscle mass, and enhance glucose sensitivity, thereby helping to prevent sarcopenia in individuals with chronic liver disease [44]. Combining post-treatment liver function assessment with SMI monitoring may serve as a valuable tool for evaluating the overall condition of patients and predicting prognosis in advanced HCC.

This study has limitations inherent to its single-center retrospective design. The proportion of female patients was relatively low, and the immunotherapy and targeted therapy groups had smaller sample sizes than the combination therapy group. Muscle strength, physical function, and ambulatory capacity were not assessed. Moreover, post-treatment, patients may have received additional anticancer therapies, introducing potential outcome bias. Future studies should validate these findings in larger populations and explore the underlying mechanisms through experimental research.

## Figures and Tables

**Table 1:** Baseline characteristics of the patients.

Characteristics	Decreased SMI	Non-Decreased SMI	
	n = 150 (49.02%)	n = 156 (50.98%)	*p* Value
Age (years): Mean ± SD	58.76 ± 11.16	60.35 ± 11.73	0.225
Gender (n): Female/Male (%)	16 (10.67)/134 (89.33)	17 (10.90)/139 (89.1)	0.954
Height (m): Median (Q_1_, Q_3_)	1.67 (1.62, 1.70)	1.67 (1.62, 1.70)	0.564
Body Weight (kg): Median (Q_1_, Q_3_)	63.75 (57.00, 72.00)	60.00 (55.00, 67.62)	0.015
BMI (kg/m^2^): Median (Q_1_, Q_3_)	23.44 (20.96, 25.31)	22.25 (20.13, 24.33)	0.011
Baseline SMI (kg/m^2^): Mean ± SD	Male:48.40 ± 8.09 Female: 39.90 ± 5.99	Male: 44.76 ± 7.09 Female: 36.16 ± 6.23	<0.001 0.089
Low Baseline SMI (n): (%)	Male: 28 (18.67) Female: 6 (4.00)	Male: 50 (32.05) Female: 12 (7.69)	0.838
Total bilirubin (umol/L): Median (Q_1_, Q_3_)	14.50 (11.00, 22.75)	13.00 (10.00, 20.00)	0.085
Albumin (g/dL): Median (Q_1_, Q_3_)	36.29 ± 4.80	36.81 ± 4.93	0.354
ALT (U/L): Median (Q_1_, Q_3_)	34.50 (23.25, 59.00)	31.00 (21.00, 54.00)	0.188
PT (s): Median (Q_1_, Q_3_)	14.10 (13.50, 14.90)	14.10 (13.40, 14.93)	0.578
CONUT score (n): Median (Q_1_, Q_3_)	3.00 (2.00, 5.00)	3.00 (1.00, 4.00)	0.158
HBV-DNA (n): (%)			0.954
≤20,000	114 (76.00)	119 (76.28)	
>20,000	36 (24.00)	37 (23.72)	
AFP (ng/mL) (n): (%)			0.906
≤400	100 (66.67)	103 (66.03)	
>400	50 (33.33)	53 (33.97)	
Hepatitis B, n (%)	48 (32.00)	45 (28.85)	0.549
ALBI grade (n): (%)			0.485
1	43 (28.67)	53 (33.97)	
2	104 (69.33)	98 (62.82)	
3	3 (2.00)	5 (3.21)	
Child–Pugh classification (n): (%)			0.754
A	109 (72.67)	119 (76.28)	
B	40 (26.67)	36 (23.08)	
C	1 (0.67)	1 (0.64)	
NLR (n): (%)			0.111
≤4	98 (65.33)	115 (73.72)	
>4	52 (34.67)	41 (26.28)	
Treatment sessions between CT exams (n): (%)			0.222
≤2	100 (66.67)	114 (73.08)	
>2	50 (33.33)	42 (26.92)	
TNM Staging LCSGJ 6th (n): (%)			0.358
I	7 (4.67)	13 (8.33)	
II	32 (21.33)	32 (20.51)	
III	70 (46.67)	81 (51.92)	
IVA	34 (22.67)	26 (16.67)	
IVB	7 (4.67)	4 (2.56)	
Maximum size of tumor (n): cm (%)			0.808
≤5	51 (34.00)	51 (32.69)	
>5	99 (66.00)	105 (67.31)	
Number of tumors (n): (%)			<0.001
≤3	83 (55.33)	115 (73.72)	
>3	67 (44.67)	41 (26.28)	
PVTT (n): (%)			0.002
YES	48 (32.00)	26 (16.67)	
NO	102 (68.00)	130 (83.33)	

BMI, body mass index; SMI, skeletal muscle mass; CONUT, Controlling Nutritional Status; ALBI, Albumin–Bilirubin score; ALT, alanine aminotransferase; PT, prothrombin time; AFP, α-fetoprotein; NLR, neutrophil-to-lymphocyte ratio; TNM, tumor–lymph node metastasis; PVTT, portal vein tumor thrombus; LCSGJ, Liver Cancer Study Group of Japan.

**Table 2:** SMI and ΔSMI after 3 months in different groups.

Measurement	TACE (n = 133)	TACE + T (n = 53)	TACE + I (n = 52)	TACE + I + T (n = 68)	*p* Value
SMI (cm^2^/m^2^)	45.09 ± 7.60	47.13 ± 8.36	44.84 ± 9.32	46.10 ± 7.81	0.372
ΔSMI (cm^2^/m^2^)	−0.02 ± 0.10	−0.05 ± 0.09	−0.03 ± 0.09	−0.05 ± 0.11	0.148

SMI, Skeletal Muscle Index; ∆SMI, Change in Skeletal Muscle Index; TACE, Transarterial Chemoembolization; I, Immunotherapy; I + T, Targeted Therapy combined with Immunotherapy; T, Targeted Therapy; *p*, *p*-value.

**Table 3:** Best therapeutic effect.

Therapeutic Effect	Decreased SMI	Non-Decreased SMI	
	n = 150	n = 156	*p* Value
ORR, n (%)	52 (34.7%)	84 (53.8%)	<0.01
DCR, n (%)	112 (74.7%)	133 (85.3%)	0.030
CR, n (%)	5 (3.3%)	18 (11.5%)	
PR, n (%)	47 (31.3%)	66 (42.3%)	
SD, n (%)	60 (40.0%)	49 (31.4%)	
PD, n (%)	38 (25.3%)	23 (14.7%)	

SMI, skeletal muscle index; ORR, overall response rate; DCR, disease control rate; CR, complete response; PR, partial response; SD, stable disease; PD, progressive disease.

**Table 4:** Univariate and multivariate analyses of tumor response.

Variables	Univariate Analysis		Multivariate Analysis	
	OR (95%CI)	*p* Value	OR (95%CI)	*p* Value
Age (years), ≥75/<75	0.94 (0.41~2.14)	0.264		
Gender, Female/Male	1.00 (0.48~2.07)	0.997		
BMI (kg/m^2^), <23/≥23	1.29 (0.82~2.03)	0.264		
Group, Decreased SMI/Non-Decreased SMI	0.38 (0.24~0.60)	<0.001	0.43 (0.26~0.70)	<0.001
SMI Group, Low-SMI Group/High-SMI Group	1.09 (0.67~1.77)	0.731		
HBVDNA, >20,000/≤20,000	1.11 (0.65~1.89)	0.700		
AFP (ng/mL), >400/≤400	0.85 (0.53~1.37)	0.498		
NLR, >4/≤4	0.59 (0.36~0.98)	0.040	0.79 (0.46~1.37)	0.403
Child–Pugh Score, ≥7/<7	0.47 (0.27~0.81)	0.007	0.53 (0.29~0.95)	0.032
ALBI Grade, >1/≤1	0.76 (0.47~1.24)	0.278		
CONUT Score, ≥2/<2	1.02 (0.60~1.74)	0.945		
Number of Tumors, >3/≤3	0.27 (0.16~0.46)	<0.001	0.35 (0.20~0.60)	<0.001
Treatment Sessions Between CT Exams, >2/≤2	1.08 (0.66~1.76)	0.762		
Maximum Tumor Size, >5 cm/≤5 cm	0.60 (0.37~0.97)	0.035	0.69 (0.40~1.19)	0.185
PVTT, YES/NO	0.57 (0.33~0.98)	0.042	1.03 (0.55~1.93)	0.922

BMI, body mass index; SMI, skeletal muscle mass; CONUT, Controlling Nutritional Status; ALBI, Albumin–Bilirubin; PVTT, portal vein tumor thrombus; AFP, α-fetoprotein; NLR, neutrophil-to-lymphocyte ratio.

**Table 5:** Univariate and multivariate analyses of OS.

Variables	Univariate Analysis		Multivariate Analysis	
	HR (95%CI)	*p* Value	HR (95%CI)	*p* Value
Age (years), ≥75/<75	1.08 (0.61~1.90)	0.792		
Gender, Female/Male	0.92 (0.56~1.52)	0.745		
BMI (kg/m^2^), <23/≥23	1.15 (0.85~1.56)	0.363		
Group, Decreased SMI/Non-Decreased SMI	1.87 (1.38~2.54)	<0.001	1.60 (1.17~2.19)	0.003
SMI Group, Low-SMI Group/High-SMI Group	1.17 (0.85~1.62)	0.325		
HBVDNA, >20,000/≤20,000	1.38 (0.97~1.96)	0.072		
AFP (ng/mL), >400/≤400	1.10 (0.80~1.51)	0.564		
NLR, >4/≤4	1.70 (1.24~2.34)	<0.001	1.24 (0.89~1.74)	0.210
Child–Pugh Score, ≥7/<7	1.40 (1.00~1.95)	0.051		
ALBI Grade, >1/≤1	1.57 (1.12~2.20)	0.009	1.18 (0.82~1.71)	0.378
CONUT Score, ≥2/<2	1.50 (1.02~2.20)	0.040	1.27 (0.83~1.95)	0.264
Number of Tumors, >3/≤3	2.33 (1.70~3.19)	<0.001	1.88 (1.35~2.62)	<0.001
Treatment Sessions Between CT Exams, >2/≤2	1.01 (0.73~1.41)	0.937		
Maximum Tumor Size, >5 cm/≤5 cm	2.10 (1.48~2.97)	<0.001	1.66 (1.15~2.40)	0.007
PVTT, YES/NO	2.03 (1.45~2.84)	<0.001	1.45 (1.01~2.07)	0.044

BMI, body mass index; SMI, skeletal muscle mass; CONUT, Controlling Nutritional Status; ALBI, Albumin–Bilirubin; PVTT, portal vein tumor thrombus; AFP, α-fetoprotein; NLR, neutrophil-to-lymphocyte ratio.

## Data Availability

The datasets presented in this article are not readily available due to ethical and privacy restrictions related to patient data. Requests to access the datasets should be directed to the corresponding author.

## Supplementary Materials

The following supporting information can be downloaded at: https://www.mdpi.com/article/10.3390/jcm15135315/s1, Table S1: AEs that occurred during the clinical course of therapy.

## Abbreviations

HCC	hepatocellular carcinoma
TACE	transarterial chemoembolization
OS	overall survival
SMI	skeletal muscle index
CT	computed tomography
L3	third lumbar vertebral level
BMI	body mass index
TNM	tumor–node–metastasis
PVTT	portal vein tumor thrombus
AFP	alpha-fetoprotein
CONUT	Controlling Nutritional Status
ALBI	Albumin–Bilirubin
JSH	Japanese Society for the Study of Hepatology
ΔSMI	change in skeletal muscle index
ROC	receiver operating characteristic
ANOVA	analysis of variance
SD	standard deviation
OR	odds ratio
HR	hazard ratio
CI	confidence interval
PD-1	programmed cell death 1
PD-L1	programmed cell death ligand 1
ICI	immune checkpoint inhibitor
TKI	tyrosine kinase inhibitor
VEGF	vascular endothelial growth factor
SIR	Society of Interventional Radiology
mRECIST	modified Response Evaluation Criteria in Solid Tumors
CR	complete response
PR	partial response
SD (response)	stable disease
PD (response)	progressive disease
ORR	objective response rate
DCR	disease control rate
HBV	hepatitis B virus
HBV-DNA	hepatitis B virus deoxyribonucleic acid
ALT	alanine aminotransferase
PT	prothrombin time
NLR	neutrophil-to-lymphocyte ratio
LCSGJ	Liver Cancer Study Group of Japan
IL-15	interleukin-15
