# Supplementary material for: Prognostic Value of Skeletal Muscle Loss in Unresectable Hepatocellular Carcinoma Treated with TACE-Based Combination Therapy
Source: J Clin Med. 2026 Jul 7;15(13):5315. doi: 10.3390/jcm15135315 (PMC13362567; doi:10.3390/jcm15135315)
Supplement: Supplementary file 1 [file jcm-15-05315-s001.zip › jcm-4345212-supplementary.pdf]

Table S1. AEs that occurred during the clinical course of therapy.

	Decreased SMI	Non-Decreased SMI	
	(n = 150)	(n = 156)	*P Value*
AEs Grade, n(%)	83(55.33)	69 (44.23)	0.381
1	29 (12.67)	22 (7.69)	
2	34 (16.00)	31 (13.46)	
3	18 (12.00)	15 (9.62)	
4	2 (1.33)	1 (0.64)	
5	0	0	
AST increased, n(%)	21 (10.67)	18 (11.54)	0.519
Anorexia, n(%)	24 (16.00)	26 (10.26)	0.875
Vomiting, n(%)	28 (12.00)	24 (8.97)	0.445
WBCs decreased, n(%)	11 (4.00)	6 (3.85)	0.183
Anemia, n(%)	3 (2.00)	1 (0.64)	0.587
Upper gastrointestinal hemorrhage, n(%)	0 (0.00)	2 (1.28)	0.499
Creatinine increased, n(%)	1 (0.67)	1 (0.64)	1.000
Hepatic failure, n(%)	1 (0.67)	0 (0.00)	0.490

AEs, Any adverse events;AST, aspartate aminotransferase; WBC, white blood cell.
